# Advanced glycation end products induce senescence of atrial myocytes and increase susceptibility of atrial fibrillation in diabetic mice

**DOI:** 10.1111/acel.13734

**Published:** 2022-10-24

**Authors:** Dan‐Lin Zheng, Qing‐Rui Wu, Peng Zeng, Sui‐Min Li, Yong‐Jiang Cai, Shu‐Zhen Chen, Xue‐Shan Luo, Su‐Juan Kuang, Fang Rao, Ying‐Yu Lai, Meng‐Yuan Zhou, Fei‐Long Wu, Hui Yang, Chun‐Yu Deng

**Affiliations:** ^1^ Guangdong Provincial Key Laboratory of Clinical Pharmacology Research Center of Medical Sciences, Guangdong Provincial People's Hospital, Guangdong Academy of Medical Sciences Guangzhou China; ^2^ Department of Cardiology, Guangdong Cardiovascular Institute Guangdong Provincial People's Hospital, Guangdong Academy of Medical Sciences Guangzhou China; ^3^ School of Medicine South China University of Technology Guangzhou China; ^4^ School of Pharmaceutical Sciences Southern Medical University Guangzhou China

**Keywords:** AGEs, atrial fibrillation, cell senescence, diabetes, electrical remodeling, p16 and Rb

## Abstract

Diabetes mellitus (DM) is a common chronic metabolic disease caused by significant accumulation of advanced glycation end products (AGEs). Atrial fibrillation (AF) is a common cardiovascular complication of DM. Here, we aim to clarify the role and mechanism of atrial myocyte senescence in the susceptibility of AF in diabetes. Rapid transesophageal atrial pacing was used to monitor the susceptibility of mice to AF. Whole‐cell patch‐clamp was employed to record the action potential (AP) and ion channels in single HL‐1 cell and mouse atrial myocytes. More importantly, anti‐RAGE antibody and RAGE‐siRNA AAV9 were used to investigate the relationship among diabetes, aging, and AF. The results showed that elevated levels of p16 and retinoblastoma (Rb) protein in the atrium were associated with increased susceptibility to AF in diabetic mice. Mechanistically, AGEs increased p16/Rb protein expression and the number of SA‐β‐gal‐positive cells, prolonged the action potential duration (APD), reduced protein levels of Cav1.2, Kv1.5, and current density of *I*_*Ca*,*L*_, *I*_*Kur*_ in HL‐1 cells. Anti‐RAGE antibody or RAGE‐siRNA AAV9 reversed these effects in vitro and in vivo, respectively. Furthermore, downregulating p16 or Rb by siRNA prevented AGEs‐mediated reduction of Cav1.2 and Kv1.5 proteins expression. In conclusion, AGEs accelerated atrial electrical remodeling and cellular senescence, contributing to increased AF susceptibility by activating the p16/Rb pathway. Inhibition of RAGE or the p16/Rb pathway may be a potential therapeutic target for AF in diabetes.

## Graphical abstract

Diabetes mellitus (DM) is a common chronic metabolic disease and an independent risk factor for atrial fibrillation (AF). However, the regulatory mechanisms underlying atrial electrical remodeling in diabetes are not fully understood. This study presents new insights into the role and mechanism of atrial myocyte senescence in diabetic‐induced AF. Inhibition of RAGE or the p16/Rb pathway may be a potential therapeutic target for AF in diabetes.

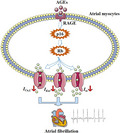

**Abbreviations**
AAV9: Adeno‐associated Virus 9
APD: Action potential duration
APD_90_
: Action potential duration at 90% repolarization
APD_50_
: Action potential duration at 50% repolarization
APA: Action potential amplitude
AF: Atrial fibrillation
AFIR: Atrial fibrillation induced rate
AGEs: Advanced glycation end products
BSA: Bovine serum albumin
CSNRT: Correct sinus node recovery time
DM: Diabetes Mellitus
EGTA: Ethylene glycol bis (2‐aminoethyl ether) tetraacetic acid
FBS: Fetal bovine serum
*I*_*Ca,L*_
: L type calcium current
MDAF: Mean duration atrial fibrillation
MEM: Minimum Essential Medium (Eagle)
PBS: Phosphate buffered solution
PDW: P wave duration
PR: PR interval
PT: PT interval
QRS: QRS wave
Rb: Retinoblastoma protein
SNRT: Recovery time of the sinus node
RAGE: Receptor of advanced glycation of end products
STZ: Streptozotocin
SCL: Sinus cycle length
SA‐β‐gal: Senescence‐associated β‐galactosidase
siRNA: Small interfering Ribonucleic Acid
SDS: Sodium dodecyl sulfate
TBS: Tris buffered saline
*I*_*to*_
: Transient outward potassium current
*I*_*Kur*_
: Ultra‐rapid delayed rectifier potassium current

## 1 INTRODUCTION

Diabetes mellitus (DM) is a common chronic metabolic disease and an independent risk factor for atrial fibrillation (AF) (Gorenek et al., 2017). The global diabetes prevalence in 2019 is estimated to be 9.3% (463 million people) and is expected to increase to 10.9% (700 million) by 2045 (Cho et al., 2018), diabetic patients with AF are also increasing dramatically. Atrial electrical remodeling has also been reported as an important pathophysiology of AF (Andrade et al., 2014) with changes in electrophysiology and ion channels (Gorenek et al., 2017; Schotten et al., 2011). Previous studies in patients and animal models have shown that electrical remodeling, especially ion channel remolding of atrial myocytes, may play a crucial role in the occurrence and maintenance of AF (Andrade et al., 2014). For example, recent studies identified significant changes in repolarizing K^+^, Na^+^, and L‐type Ca^2+^ currents along with impaired Ca^2+^ homeostasis and defective contractile function in the diabetic heart (Ozturk et al., 2021). A higher incidence of inducible AF was observed in ZDF rats due to the decreased conduction velocity and ion channel function (Fu et al., 2019). However, the regulatory mechanisms underlying atrial electrical remodeling in diabetes are not fully understood.

Cellular senescence, an unavoidable process in all living organisms, is a key characteristic of individual aging processes. Classically, senescence involves the p53 and p16/Rb pathways, both of these tumor suppressor pathways need to be abrogated to avoid senescence (Campisi & d'Adda di Fagagna, 2007). A recent study elucidates that excessive and aberrant accumulation of senescent cells in different cardiac cell types can be positively associated with a risk of cardiovascular diseases (CVD) such as atherosclerosis, myocardial infarction, and cardiac fibrosis (Chen et al., 2022). Accumulating evidence has indicated that AF progression is strongly related to human atrial senescence accompanied by upregulation of p53 and p16 proteins, suggests a link between senescence and atrial remodeling (Jesel et al., 2019; Kazemian et al., 2012). On the contrary, DM is considered an inducer of accelerated cellular senescence and has been related to aging‐related cardiovascular diseases because of high glucose levels (Gu et al., 1998). This epidemiological connection between aging, DM, and AF suggests a pathophysiological link (Shakeri et al., 2018).

Advanced glycation end products (AGEs), combined with the receptor for advanced glycation end products (RAGE) are increased dramatically and involved in the pathogenesis of CVD (Méndez et al., 2010). RAGE is a member of the immunoglobulin superfamily of cell surface proteins that bind AGEs and other molecules. It has been demonstrated that AGEs and RAGE are accumulated in various organs in diabetes such as pancreas and heart, as well as nephropathy (Guo et al., 2020; Li et al., 2010; Martins et al., 2016; Palmer et al., 2019). RAGE may be produced as different isoforms, full length RAGE (FL‐RAGE), endogenous soluble RAGE (es‐RAGE), and soluble RAGE (sRAGE) resulting from proteolytic cleavage by ADAM10 (Haddad et al., 2016). Several theories have been published according to the equilibrium between the different isoforms, the soluble RAGE and anti‐RAGE antibody are possibly protective by preventing the interaction of AGEs to cellular RAGE (Qi & Ma, 2017). Additionally, AGEs play a crucial role in cardiomyocyte senescence by upregulating expression level of the p16 protein, an increase in the number of SA‐β‐gal‐positive cells has also been observed (Zha et al., 2017). Some studies have shown that AGEs restore the adipogenic potential of senescent preadipocytes through modulation of the p53/p21 pathway (Chen et al., 2012). However, the related mechanism of AGEs in mediating diabetic AF remains elusive. The present study aims to determine the role and mechanism of AGEs in electrical remodeling of atrial myocytes and enhanced AF susceptibility in diabetic mice.

## 2 RESULTS

### 2.1 Increased susceptibility to AF in diabetic mice

**FIGURE 1:**
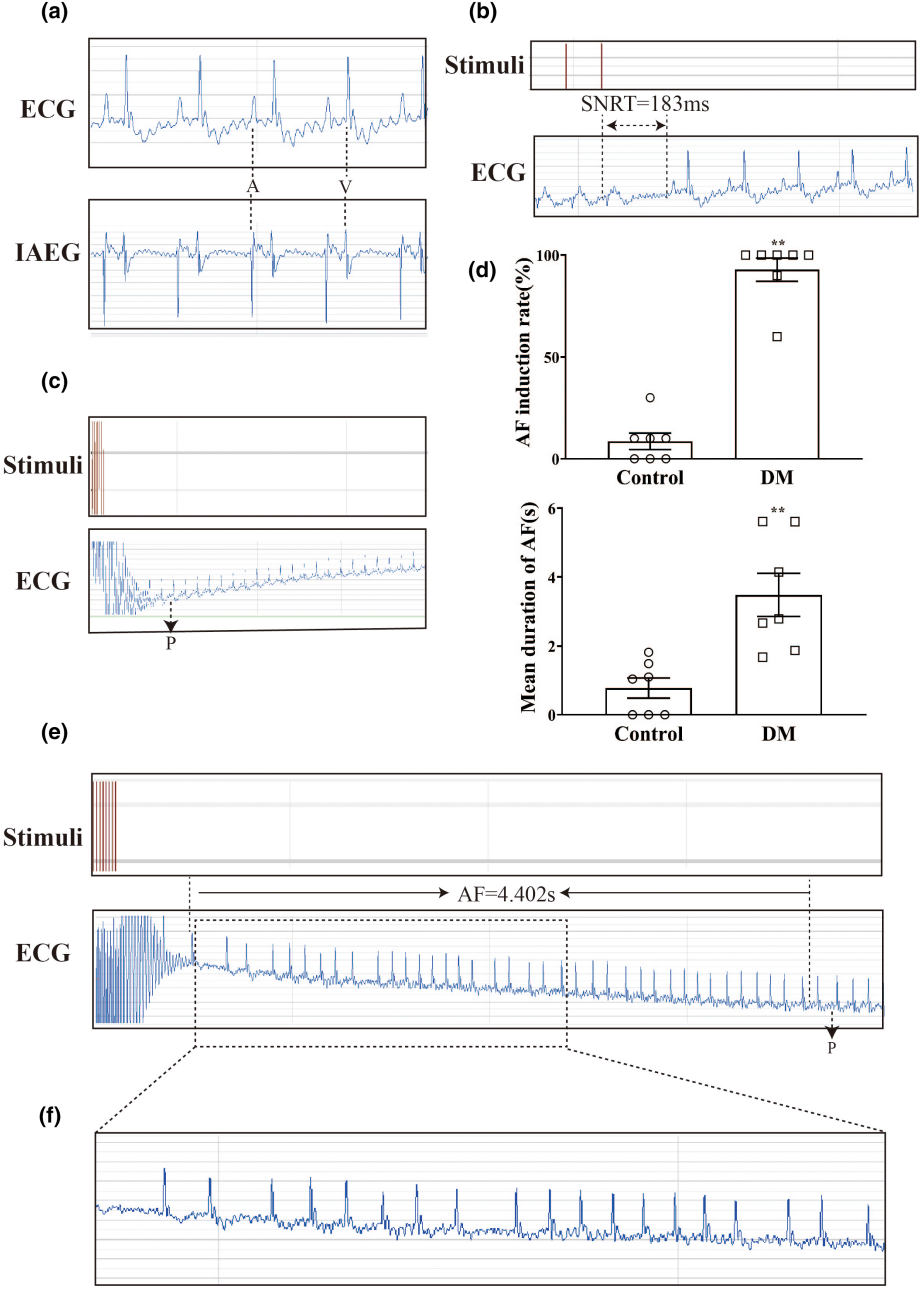
Representative electrophysiology results for atrial rapid pacing in diabetic mice. (a) Typical baseline surface electrocardiogram (ECG) and intra atrial electrocardiogram (IAEG). (b) Typical surface ECG recordings of sinus node recovery time (SNRT) following a 6 s pacing train. (c) Typical surface ECG recordings of rats maintaining SR after 15 s of atrial burst pacing. (d) AF induction rate and mean duration of AF in Control and DM mice (*n* = 7). (e) Typical surface ECG recordings of diabetic mice with AF that spontaneously reverted to SR. (f) Typical disorganized atrial wave (f wave). ^**^ *p* < 0.01 vs. Control group.

Rapid transesophageal atrial pacing was used to evaluate susceptibility to AF in Control and Diabetic groups (Figure 1). Notably, the incidence rate of atrial fibrillation (AFIR) in diabetic mice was higher, and the mean duration of atrial fibrillation (MDAF) was longer than that in the Control group (Figure 1d, Table S1). A significant increase was observed in P‐wave duration (PWD) and RR intervals in diabetic mice. However, SNRT, CSNRT, PR, QRS, and QT did not differ between the two groups (Table S1). Thus, diabetic mice are more likely to develop AF than control mice.

### 2.2 Alterations in atrial myocyte electrophysiology of diabetic mice

**FIGURE 2:**
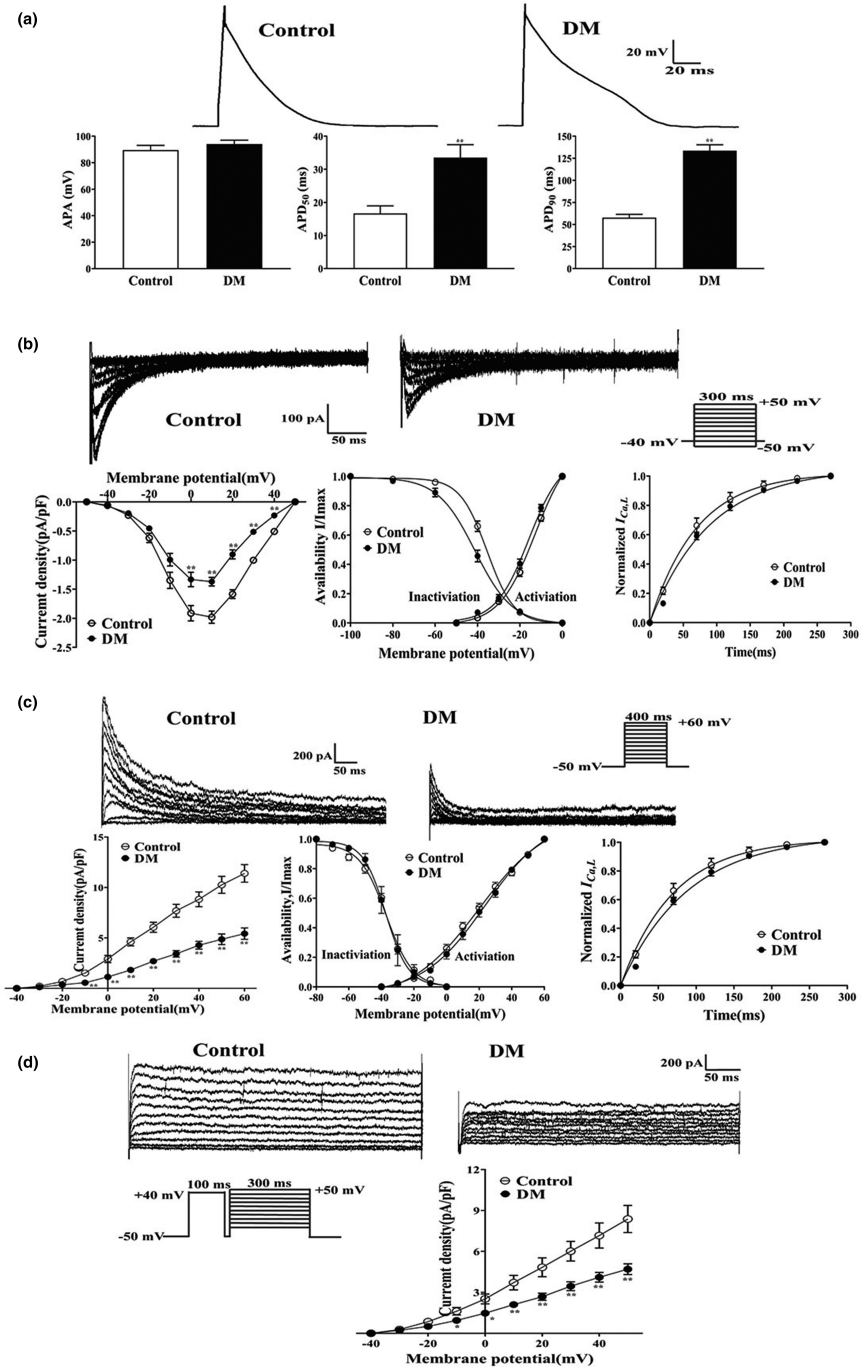
Effects of APD and ion channel currents in atrial myocytes of diabetic mice. (a) Representative traces of APD [*n* = 9–11/6 (myocytes/mice)] in atrial myocytes from Control and DM groups. (b) Representative traces (pulse protocol, inset), corresponding current–voltage (*I*‐*V*) relationship, mean data for voltage dependence activation, inactivation, and time course of recovery current for *I*_*Ca*,*L*_ [*n* = 9–16/6 (myocytes/mice)] in atrial myocytes from Control and DM mice. (c) Representative traces (pulse protocol, inset), corresponding current–voltage (*I*‐*V*) relationship, mean data for voltage dependence activation, inactivation, and time course of recovery current for *I*_*to*_ [*n* = 8–12/6 (myocytes/mice)] in atrial myocytes from Control and DM mice. (d) Representative traces and current–voltage (*I*‐*V*) relationship for *I*_*Ku*r_ [*n* = 8–9/6 (myocytes/mice)] in atrial myocytes from Control and DM mice. ^**^ *p* < 0.01 vs. Control group.

The electrical remodeling of diabetic atrial myocytes was also explored. An alteration of action potential duration (APD) in atrial myocytes was recorded in both groups. The action potential amplitude (APA) and APD at 50%, 90% repolarization (APD_50_, APD_90_) were elicited in a whole‐cell current‐clamp mode. Compared with the Control group, no significant change was observed in the APA of atrial myocytes from diabetic mice (*n* = 9–10, *p* > 0.05). However, APD_50_ and APD_90_ were significantly prolonged in dissociated atrial myocytes from diabetic mice (*n* = 10–11, *p* < 0.01) (Figure 2a, Table S2).

To explore the specific mechanism of action potential prolongation in atrial myocytes of diabetic mice, ion channel currents were measured using a whole‐cell voltage‐clamp mode. The peak current of *I*_*Ca*,*L*_ was reduced in diabetic mice compared with the control mice (−1.97 ± 0.10 pA/pF in Control group vs. ‐1.37 ± 0.08 pA/pF in DM group, *n* = 9–11, *p* < 0.01), while the activation potential, peak potential, and reversal potential were not significantly different. No significant differences were observed in the activation and recovery kinetics of *I*_*Ca*,*L*_ in diabetic mice compared with the control mice (*n* = 10–12, *p* > 0.05), but the inactivation curve was significantly shifted to the left (*n* = 15–16, *p* < 0.01) (Figure 2b, Table S2). The current densities of Ito and IKur were substantially lower in atrial myocytes from diabetic mice than from the controls (11.39 ± 0.87 pA/pF vs. 5.43 ± 0.56 pA/pF, at 60 mV; 8.37 ± 0.99 pA/pF vs. 4.83 ± 0.51 pA/pF, at 50 mV; for *I*_*to*_ and *I*_*Kur*_, respectably, *n* = 8–12, *p* < 0.01) (Figure 2c and d).

### 2.3 Alterations of ion channels, AGE, RAGE, and p16/Rb proteins expression in atrium of diabetic mice

**FIGURE 3:**
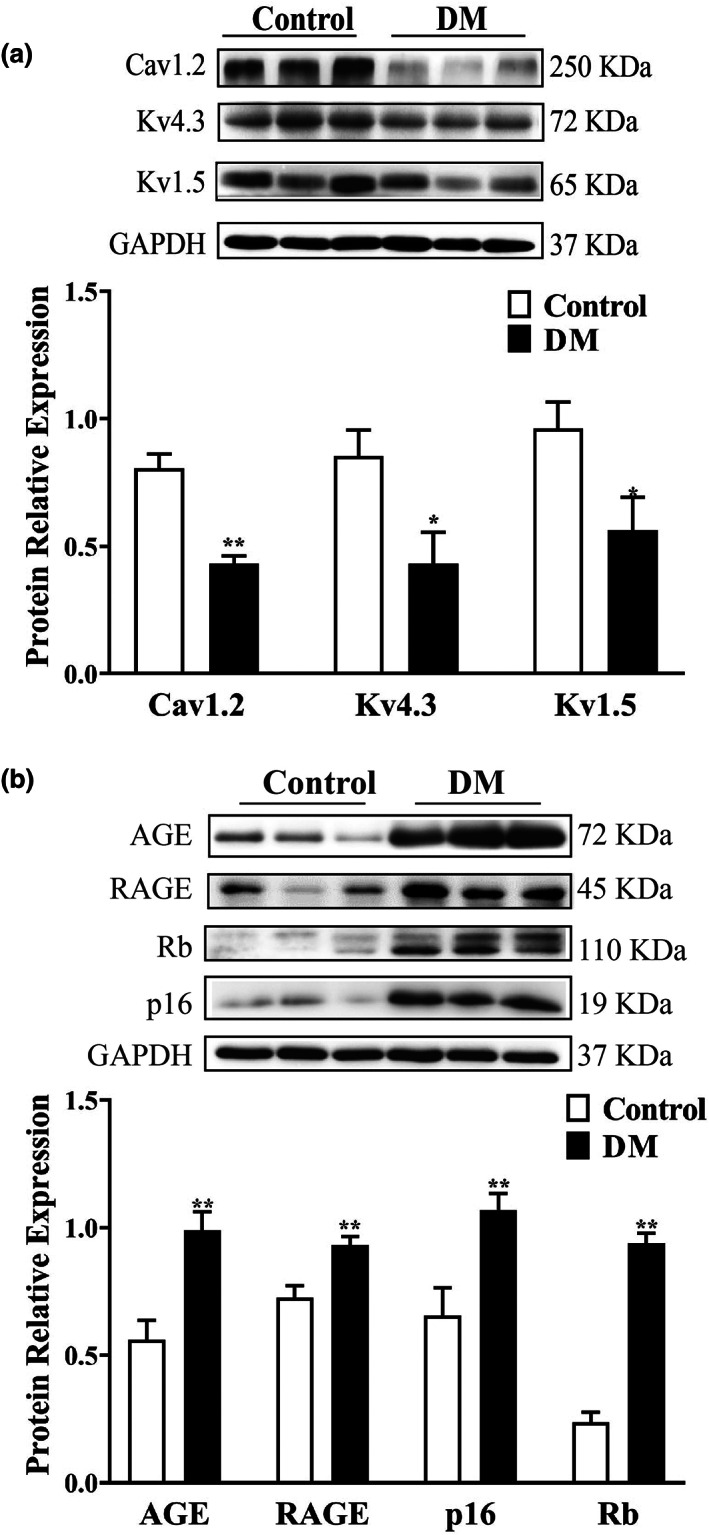
Expression of ion channels, AGE, RAGE and p16/Rb proteins in atrium of diabetic mice. (a) Representative blots and densitometry analysis of Cav1.2, K4.3, and Kv1.5 proteins in atrial tissues from Control and DM groups (*n* = 7–12). (b) Representative blots and densitometry analysis of AGE, RAGE, p16, Rb proteins in atrial tissues from Control and DM groups (*n* = 6–12). *p < 0.05, **p < 0.01 vs. Control group.

Next, we detected the expression of ion channels, AGE, RAGE, and p16/Rb proteins in atrium of diabetic mice. Consistent with a reduction in current density, Cav1.2, Kv4.3, and Kv1.5 protein levels were significantly reduced in atrial tissues of diabetic mice (*n* = 7–12, *p* < 0.05) (Figure 3a). Compared with the control mice, expression levels of AGE and RAGE, p16 and Rb proteins were significantly increased in atrial tissue from diabetic mice (*n* = 6–12, *p* < 0.05) (Figure 3b), suggesting the AGEs/RAGE pathways and p16/Rb pathways were up‐regulated in diabetic mice.

### 2.4 Effects of AGEs/RAGE on electrical remodeling in atrial myocytes

**FIGURE 4:**
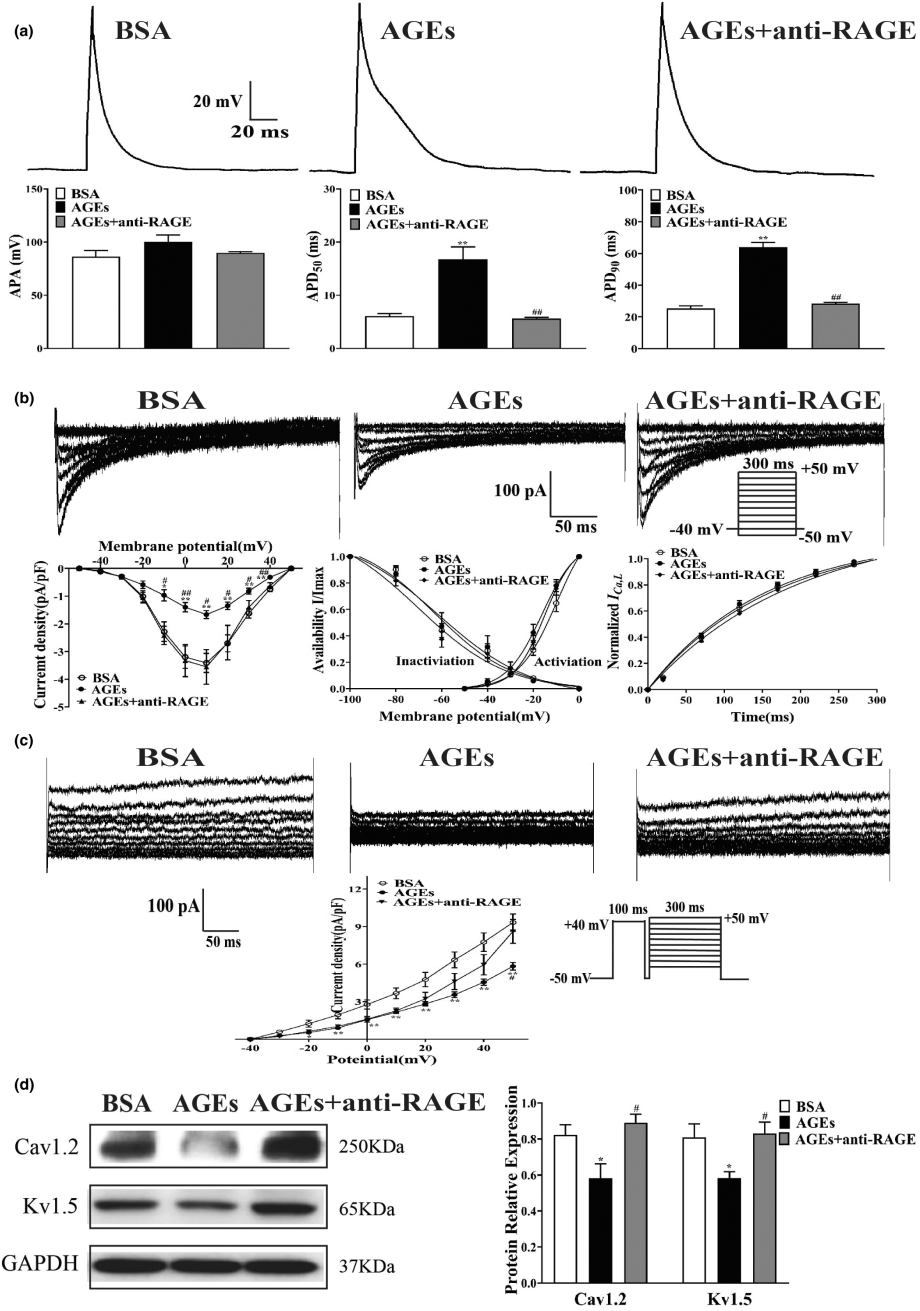
Effects of APD and ion channel currents on HL‐1 cells treated with AGEs or anti‐RAGE antibody. (a) Representative traces of APD (*n* = 10–12) in HL‐1 cells treated with AGEs and anti‐RAGE antibody. (b) Representative traces (pulse protocol, inset), corresponding current–voltage (*I*‐*V*) relationship, mean data for voltage dependence activation, inactivation, and time course of recovery current for *I*_*Ca*,*L*_ (*n* = 6–12) in HL‐1 cells treated with AGEs or anti‐RAGE antibody. (c) Representative traces and current–voltage (*I*‐*V*) relationship for *I*_*Ku*r_ (*n* = 5–8) in HL‐1 cells treated with AGEs or anti‐RAGE antibody. (d) Representative blots and densitometry analysis of Cav1.2 and Kv1.5 proteins in HL‐1 cells treated with AGEs or anti‐RAGE antibody (*n* = 4). *p < 0.05 vs. BSA group. ^#^ *p* < 0.05 vs. AGEs group.

To evaluate the changes in APD in HL‐1 cells treated with AGEs, the APA and APD_50_, APD_90_ were assessed in a current clamp mode. Both APD_50_ and APD_90_ of HL‐1 cells induced by AGEs were significantly prolonged compared with the BSA group. In addition, inhibition of RAGE by anti‐RAGE antibody inhibited this effect (*n* = 10–12, *p* < 0.01). However, there were no changes in APA among the three groups (*n* = 10–12, *p* > 0.05) (Figure 4a, Table S3).

The current density of *I*_*Ca*,L_ was decreased significantly in HL‐1 cells following treatment with AGEs (−3.29 ± 0.39 pA/pF in BSA group vs. ‐1.66 ± 0.15 pA/pF in AGEs group, *n* = 7–9, *p* < 0.01). This effect was alleviated by anti‐RAGE antibody (−1.66 ± 0.15 pA/pF in AGEs group vs. ‐3.55 ± 0.63 pA/pF in AGEs + anti‐RAGE group, *n* = 7, *p* < 0.05) (Figure 4b). There were no differences in the activation, inactivation, and recovery curves of *I*_*Ca*,*L*_ among the three groups (Table S3).

*I*_*Kur*_ was also recorded in HL‐1 cells treated with AGEs and anti‐RAGE antibody. Figure 4c showed that the current density of *I*_*Kur*_ was significantly reduced in HL‐1 cells after AGEs treatment compared with the BSA group (9.33 ± 0.69pA/pF in BSA group vs.5.83 ± 0.31pA/pF in AGEs group, at 50 mV, *n* = 7–8, *p* < 0.01). This effect could be reversed by treatment with anti‐RAGE antibody (5.83  ±  0.31 pA/pF in AGEs group vs.8.62  ±  0.96pA/pF in AGEs  ±  anti‐RAGE group, at 60 mV, n = 5–8, *p* < 0.01).

The protein levels of Cav1.2 and Kv1.5 in HL‐1 cells treated with AGEs were detected by Western blotting. Consistent with the current density of *I*_*Ca*,*L*_ and *I*_*Kur*_, protein levels of Cav1.2 and Kv1.5 were also diminished following AGEs treatment (*n* = 4, *p* < 0.05). Importantly, this effect was reversed by anti‐RAGE antibody treatment (*n* = 4, *p* < 0.05) (Figure 4d). In general, these results corroborate preceding studies, further confirming *I*_*Ca*,*L*_ and *I*_*Kur*_ participate in the pathogenesis of atrial electrical remodeling in diabetes.

### 2.5 AGEs induce cellular senescence through the p16/Rb pathways

**FIGURE 5:**
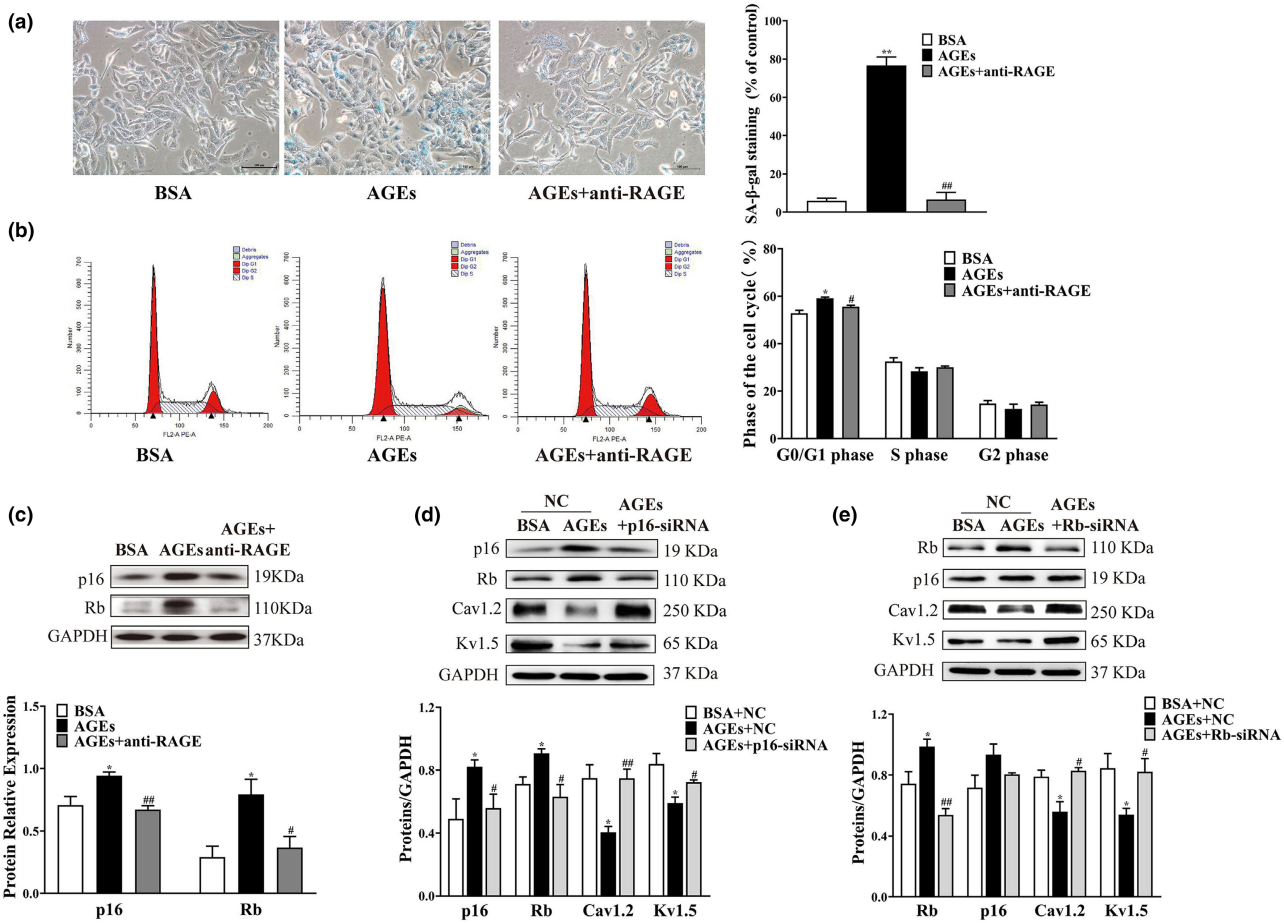
Alterations in senescence phenotype and expression levels of HL‐1 cells treated with AGEs, anti‐RAGE antibody, plasmid transfection. (a) SA‐β‐gal staining was used to elevate the positive rate of senescent cells treated with AGEs or anti‐RAGE antibody (*n* = 4). (b) Flow cytometry was used to detect cell cycle distribution in HL‐1 cells treated with AGEs or anti‐RAGE antibody (*n* = 4). (c) Representative blots and densitometry analysis of p16 and Rb proteins in HL‐1 cells treated with AGEs or anti‐RAGE antibody (*n* = 4). (d) Representative blots and densitometry analysis of Cav1.2 and Kv1.5 proteins in HL‐1 cells intervene with p16 protein (*n* = 3). (e) Representative blots and densitometry analysis of Cav1.2 and Kv1.5 proteins in HL‐1 cells intervene with Rb protein (*n* = 3–5). *p < 0.05, **p < 0.01 vs. BSA group or BSA + NC group. ^#^ *p* < 0.05, ^##^ *p* < 0.01 vs. AGEs group or AGEs + NC group.

We then used SA‐β‐Gal staining and flow cytometry to determine the relationship between diabetes and senescence in HL‐1 cells. The rate of cell senescent numbers and DNA contents in G1 phase was significantly higher in the AGEs group than in the BSA group, and the effects of AGEs could be offset by anti‐RAGE antibody treatment (*n* = 4, *p* < 0.05) (Figure 5a and b). In alignment with animal results, protein levels of p16 and Rb were elevated in HL‐1 cells treated with AGEs in comparison with the Control group. These changes were blocked by anti‐RAGE antibody, which had little effect on the Control group (*n* = 4, *p* < 0.01) (Figure 5c). These results suggest that activation of RAGE is involved in AGEs‐induced cellular senescence in diabetes.

To further confirm the mediation of senescence and atrial electrical remodeling by AGEs through the p16/Rb pathway, small interfering RNA (siRNA) was used to knockdown p16 or Rb. Western blotting showed that compared with the AGEs group, Rb protein expression was decreased in the AGEs + p16‐siRNA group (*n* = 3, *p* < 0.05). No significant change in the level of p16 protein after knockdown of Rb was observed in HL‐1 cells (*n* = 3–5, *p* > 0.05). Furthermore, expression levels of Cav1.2 and Kv1.5 proteins were significantly increased after knockdown of p16 or Rb in HL‐1 cells treated with AGEs (*n* = 3–5, *p* < 0.05) (Figure 5d, e). These results demonstrate that the activation of the p16/Rb pathway might promote atrial electrical remodeling in diabetes.

Then, we further investigated the therapeutic role of RAGE intervention in diabetic mice. As shown in Figure S2, Table S4, RAGE‐siRNA‐AAV9 injection obviously reduced the incidence rate of atrial fibrillation (AFIR) and the mean duration of atrial fibrillation (MDAF) in diabetic mice (*n* = 4–5, *p* < 0.01). Furthermore, PWD, RR intervals, SNRT, CSNRT, PR, QRS, and QT were recorded between Control, DM + Vector and DM + RAGE‐siRNA‐AAV9 groups. A significant increase was observed in CSNRT compared with the control groups, while a prominent decrease was observed in SCL, SNRT, and CSNRT in diabetic mice after RAGE‐siRNA‐AAV9 injection. However, PWD, PR, QRS, and QT did not differ among the three groups (Table S4). Meanwhile, the knockdown of RAGE also reversed the downregulation of Cav1.2, Kv1.5 and the upregulation of p16, Rb in diabetic mice (*n* = 4–6, *p* < 0.01) (Figure S3). Thus, intervention of AGEs/RAGE could alleviate the inducible of AF in diabetes.

## 3 DISCUSSION

This study presents new insights into the role and mechanism of atrial myocyte senescence in diabetic‐induced AF. In the present study, we find out that the increased susceptibility of diabetic mice to AF is associated with the decrease of *I*_*Ca*,*L*_, *I*_*to*_ and *I*_*Kur*_. Upregulation of AGEs/RAGE contributes to the remodeling of these ion channels through activating p16/Rb signaling pathway. Inhibition of AGEs/RAGE and downstream p16/Rb signaling pathway alleviated the electrophysiological remodeling of atrial myocytes and the inducible rate of AF in diabetic mice.

Diabetes is an established risk factor for AF, and atrial electrical remodeling is an important mechanism in the occurrence and maintenance of AF. Particularly, progressive electrical remodeling of cardiac ion channels is an electrophysiologic hallmark of AF. At present, it is generally believed that atrial conduction heterogeneity and APD alteration were observed in diabetic‐induced AF (Patti et al., 2017). Consistent with previous work, our findings demonstrated that the APD_50_ and APD_90_ of atrial myocytes from diabetic mice were significantly longer, while no changes in resting potential were observed. These findings suggest that ion channels of atrial myocytes are changed in DM.

Ion channels may constitute a promising therapeutic target in complications of diabetic arrhythmias (Kim et al., 2017). Previous studies have found that drug or transgenic induction of diabetic models manifested the conduction of atrial myocytes were slowed while Ca^2+^ regulation was impaired. However, the variation in *I*_*Ca*,*L*_ of atrial myocytes in diabetes remains obscure (Liu et al., 2012; Pan et al., 2016; Polina et al., 2020), although the reduction in *I*_*Ca*,*L*_ and Cav1.2 protein may be the basis of atrial electrical remolding in patients with chronic AF (Barana et al., 2014). Additionally, several types of potassium currents are activated during the action potential in atrial myocytes, including transient outward K^+^ current (*I*_*to*_) and the ultra‐rapid delayed rectifier K^+^ current (*I*_*Kur*_). Recent studies have shown that *I*_*to*_ and *I*_*Kur*_ of atrial myocytes were decreased in patients with chronic AF (Workman et al., 2001). However, few studies have reported the relationship between diabetes and K^+^ remodeling of atrial myocytes. Our results showed that *I*_*Ca*,*L*_, *I*_*to*_, and *I*_*Kur*_ current density and Cav1.2, Kv4.3, and Kv1.5 proteins were significantly downregulated, indicating that the occurrence of AF in diabetic mice may be closely associated with a decrease in calcium and potassium currents. Interestingly, the kinetics of the *I*_*Ca*,*L*_ inactivation shift to left in diabetic mice also indicates that the ion channel dynamics function is participated in AF.

Previous studies have shown that levels of AGE in diabetes are increased significantly (Chua et al., 2020). Diabetes mellitus is an aging‐related disease and the average life of a diabetic patient is shortened by 6 years compared with normal individuals. Recent studies have shown that the AGEs/RAGE pathway is involved in the senescence of neonatal rat cardiomyocytes by activating the pink1/parkin pathway (Zha et al., 2017). Protein levels of p16 and Rb have been shown to be increased in vascular endothelial cells in diabetes (Katsuumi et al., 2018; Palmer et al., 2019), while other studies have shown that accelerated senescence of ventricular myocytes in diabetic rats was related to activation of the p53/p21 pathway (Gu et al., 2018). Here, we found that protein expression of AGE, RAGE, p16, and Rb is upregulated in atrial tissue of diabetic mice, although there was no difference with p53 and p21. This finding indicates that AGEs/RAGE and p16/Rb activation may be associated with the senescence of atrial myocytes in diabetes.

Prior studies revealed that the APD was prolonged and small conductance‐activated potassium channels were decreased in HL‐1 cells induced by high glucose, which led to arrhythmia and AF (Yi et al., 2015). Our results showed that AGEs induced a downregulation of *I*_*Ca*,*L*_ and *I*_*Kur*_ current density in HL‐1 cells, while anti‐RAGE antibody significantly inhibited this phenomenon. In addition, knockdown of RAGE also reduced the incidence rate of atrial fibrillation (AFIR) in diabetic mice. p16 was found to be highly expressed in senescent cells, and it can be clear by preventing the function of the heart K_ATP_ channel to mitigate deterioration of heart senescence (Baker et al., 2016). Knockdown of p16 protein could promote stem cell proliferation and tissue regeneration as well as alleviate senescence(Kim et al., 2019). Knockout of Rb and Meis2 may lead to cell cycle re‐entry of adult cardiomyocytes and promote heart repair after myocardial infarction (Alam et al., 2019; Janzen et al., 2006). Furthermore, we confirmed that activation of RAGE participates in AGEs‐induced cell senescence in diabetes. Our study reveals that AGEs increased p16 and Rb expression, which could be reversed by inhibition or knockdown of RAGE. Therefore, we confirmed that AGEs/RAGE not only contribute to cell senescence by activating the p16/Rb pathway, but also contribute to the AF susceptibility in diabetes.

To further clarify the role of p16 and Rb in regulating ion channel function in atrial myocytes in diabetes, we knocked down p16 and Rb using siRNA technology in HL‐1 cells treated with AGEs. Downregulation of p16 or Rb protein improved ion channel function in HL‐1 cells induced by AGEs, which initially revealed that cell senescence is involved in the occurrence of AF in diabetes. For a novel perspective, this research shows that p16 or Rb protein may be a target to provide new treatment directions for diabetic induced‐AF.

**FIGURE 6:**
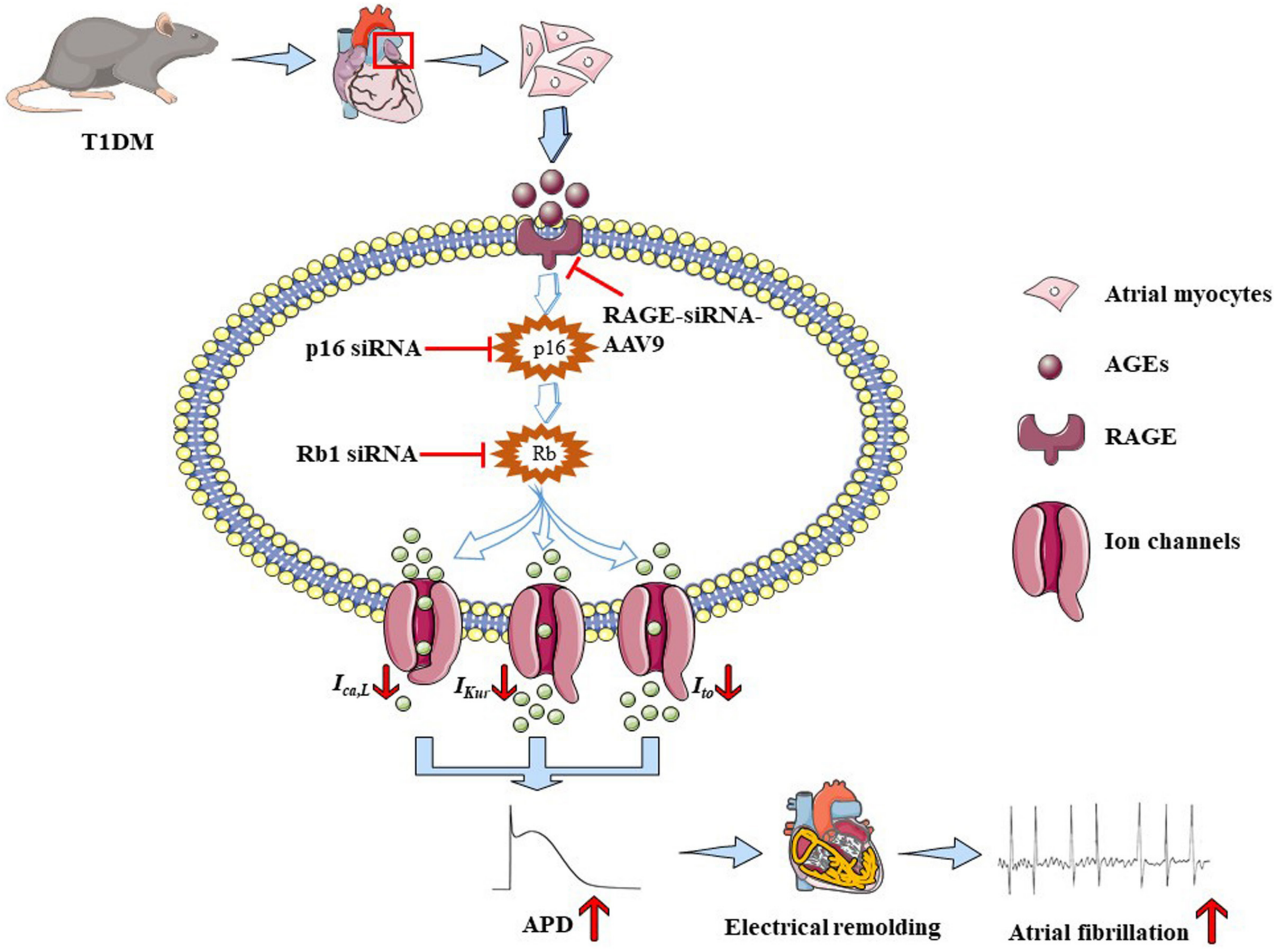
Schematic diagrams depicting proposed the mechanism of AGEs can accelerate cellular senescence of atrial myocytes through the p16/Rb pathways and increase the susceptibility to atrial fibrillation in diabetes. AGE and RAGE are accumulated in diabetes mellitus, which can accelerate cellular senescence and electrical remodeling of atrial myocytes through the p16/Rb signaling pathway. Meanwhile, reducing *I*_*Ca*,*L*_, *I*_*to*_ and *I*_*Kur*_ current density, extending the APD, and finally leading to atrial fibrillation. Mechanically, inhibition of RAGE can improve cellular senescence and atrial electrical remodeling; what is more, silencing p16 or Rb protein will promote atrial electrical remodeling by increasing the level of Cav1.2 and Kv1.5 proteins, revealing that RAGE or the p16/Rb pathway may be a potential therapeutic target for AF in diabetes.

In summary, the upregulation of AGEs/RAGE plays an important role in the inducibility of AF in diabetic mice. AGEs/RAGE accelerated the senescence of atrial myocytes through activating p16/Rb signaling pathway, then promoted the remodeling of Cav1.2, Kv4.3, and Kv1.5, prolonged the APD, resulting in the electrical remodeling of the atrium. These findings suggest that targeting the AGEs/RAGE or p16/Rb pathway may offer a promising approach to ameliorate DM‐related AF (Figure 6).

## 4 EXPERIMENTAL PROCEDURES

### 4.1 Construction of animal models

The C57BL/6 mice (8‐week‐old), male, were purchased from the GemPharmatech Co., Ltd (Nanjing, China) and GemPharmatech Co., Ltd. Mice were randomly divided into two groups (Control and DM groups) and raised in the Experimental Animal Center of South China University of Technology. After a week of acclimatization, mice were fasted for 12 h and then intraperitoneally injected with Streptozotocin(STZ, 50 mg/kg, Sigma‐Aldrich) or citric acid‐sodium citrate buffer for 5 successive days. Mice (4‐week‐old) were randomly divided into three groups (Control, DM + Vector and DM + RAGE‐siRNA‐AAV9 groups) and raised in the Experimental Animal Center of Guangdong medical laboratory. After a week of acclimatization, mice in DM group were injected 1 × 10^11^ v.g. AAV9‐Vector or AAV9‐siRAGE (Shanghai Jikai Gene Chemical Technology Co., Ltd, Shanghai, China) through tail vein for 4 weeks. The sequences of RAGE‐siRNA AAV9 used in this study were shown in Table S5. Subsequently, mice received five consecutive daily intraperitoneal injections of STZ to induce diabetes. A week after the injection of STZ, diabetic mice displayed the characteristics of “polydipsia, polyphagia, polyuria as well as loss weight”, and the random blood glucose level measured was significantly increased than control group (≥ 11.1 mmol/L), indicating that type 1 diabetic mice were successfully established (Figure S1). After accumulation at 20 weeks, mouse cardiac function was evaluated with electrophysiological measurements. All animal experiments were approved by the animal experiment ethical review committee of Guangdong Provincial People's Hospital (No. GDREC201208A).

### 4.2 Electrophysiology in vivo

Electrophysiological measurements were performed in mouse after anesthesia with 1% pentobarbital sodium (10 mg/kg) intraperitoneally. The body temperature was maintained at 37 ± 0.5°C with a heating pad (RWD Life Science Inc, Shenzhen, China) during experiment.

#### 4.2.1 Electrocardiogram (ECG) recording

The surface ECG was recorded using the iWorx Data acquisition and analysis system (iWorx Systems, Dover NH). P‐wave duration (PWD), PR interval, QRS duration, and QT interval from ten consecutive beats were measured manually.

#### 4.2.2 Sinus node function

Sinus node recovery time (SNRT) was calculated following a 6 s pacing train continuously with a BCL of 100 ms, a stimulus amplitude of 2‐fold diastolic capture threshold and a pulse width of 1 ms. SNRT was known as time period between the last stimulus and the first intrinsic atrial signal. Sinus cycle length (SCL) was determined by averaging 10 consecutive R‐R intervals. Rate corrected SNRT (CSNRT) was defined as the SCL subtracted from the SNRT.

#### 4.2.3 AF inducibility

For induction of AF, the stimulation was delivered at 15 V for a duration of 10s atrial burst pacing at a 2‐fold diastolic capture threshold (BCL: 20 ms; pulse width: 2 ms), the induction was repeated 10 times. AF was considered to be induced successfully if a period of rapid and fragmented atrial electrograms with irregular AV‐nodal conduction and ventricular rhythm persisted at least 1 s, the AF duration was defined as the interval between initiation and spontaneous termination of AF, and AF inducibility was determined by calculating the number of AF episodes and the total duration of AF divided by the number of total procedures.

### 4.3 Isolation of mouse atrial myocytes

Atrial myocytes were enzymatically isolated from atrial tissue of mouse (Fu et al., 2019). In brief, the heart was quickly removed and mounted on a Langendorff apparatus under the stereomicroscope, perfused in modified Minimum Essential Medium Eagle (MEM) solution for 10 min, and then digested for 30 min in a MEM solution containing collagenase II (CLS II) and bovine serum albumin (BSA, Sangon Biotech, China). Cells were collected and maintained at 4°C and used for experiment within 6 h after being suspended in KB solution containing (in mM): K‐Glutamate 50, KOH 20, KCl 40, Taurine 20, KH_2_PO_4_ 20, MgCl_2_·6H_2_O 3, Glucose·H_2_O 10, EGTA 0.5, HEPES 10, and pH 7.4 with KOH.

### 4.4 HL‐1 cell culture

HL‐1 cells were obtained from the laboratory of Dr. William Claycomb (Louisiana State University Health Science Center, New Orleans, LA). Cells were cultured in Claycomb medium (51,800C; Sigma‐Aldrich), supplemented with 10% fetal bovine serum (FBS) (F8687; Sigma), 2 mmol/L L‐glutamine (25,030,081; Gibco), 100 μmol/L norepinephrine (HY‐137,158; Sigma) on flasks precoated with 5 μg/ml fibronectin (356,008; Corning) and 0.02% gelatin (G‐9382; Sigma), then incubate at 37°C in a humidified atmosphere of 5% CO_2_.

AGEs‐BSA were purchased from Abcam (ab51,995, UK), which was prepared by reacting bovine serum albumin with glycolaldehyde under sterile conditions as described previously. Cells were treated with drugs when the cell fusion reached about 60%–70%. HL‐1 cells were incubated with Claycomb medium plus 10% FBS in the presence of 2 μg/ml anti‐RAGE antibody for 3 h, and then 400ug/ml AGEs‐BSA was added and cultured for 48 h, BSA (2221‐BSA, BioVision) served as the negative control group.

### 4.5 Whole‐cell patch‐clamp recording

Cells were adhered to the bottom of dish and observed using an inverted microscope (Olympus IX70, Tokyo, Japan). After cells were perfused with extracellular solution, a whole‐cell voltage‐clamp technique was used to record the L‐type calcium current (*I*_*Ca*,*L*_), the instantaneous outward potassium current (*I*_*to*_), the ultra‐rapid delayed rectify potassium current (*I*_*Kur*_), and membrane capacitance (Cm), while a current clamp was used for measuring a single cell action potential (AP).

For *I*_*Ca*,*L*_ measurements, the internal solution contained (in mM): CsCl 100, TEA‐Cl 20, Na_2_‐ATP 5, Na_2_GTP 0.4, EGTA 10, and HEPES 10, pH 7.2 (Tris). The external solution contained (in mM): Choline‐Cl 126, CsCl 5.4, MgCl_2_·6H_2_O 1, NaH_2_PO_4_·2H_2_O 0.33, Glucose·H_2_O 10, HEPES 10, and CaCl_2_·2H_2_O 2, pH 7.4 (CsOH). For *I*_*to*_ and *I*_*Kur*_ measurements, the internal solution included (in mM): KCl 140, MgCl_2_·6H_2_O 1, HEPES 10, EGTA 5, and Na_2_‐ATP 5, pH 7.2 (KOH). The external solution included (in mM): NaCl 136, KCl 5.4, MgCl_2_·6H_2_O 1, NaH_2_PO_4_·2H_2_O 0.33, HEPES 10, CaCl_2_·2H_2_O 10, CdCl_2_ 0.3, BaCl_2_ 0.5, and Glucose•H_2_O 50, pH 7.4 (NaOH). For AP measurements, the internal solution contained (in mM): KCl 140, MgCl_2_·6H_2_O 1, HEPES 10, EGTA 5, and Na_2_‐ATP 5 (pH 7.2, KOH). The external solution contained (in mM): NaCl 136, KCl 5.4, MgCl_2_·6H_2_O 1, D‐glucose 10, HEPES 10, 1.8 CaCl_2_, and NaH_2_PO_4_·2H_2_O 0.33 (pH 7.4, NaOH).

Patch pipettes were fabricated from borosilicate glass capillaries (7740, 1.6 mm OD, Corning) using a P‐97 puller (Sutter Instrument). Tip resistances were typically 2–4 MΩ when filled with the internal solution. Tip potentials were compensated before the pipette touched the cell. After a gigaseal was obtained, the cell membrane was ruptured by gentle suction to establish the whole‐cell configuration. Current signals were recorded with a MultiClamp 700B amplifier using the Digidata 1440A low noise data acquisition system (Axon Instruments). Signals were filtered at 5 kHz and stored on a computer. Series resistances (Rs) were 3–5 MΩ and were electrically compensated by 70%–80% to minimize the capacitive surge on the current recording and voltage drop across the clamped membrane and were maintained at a constant value during the current recording. Data acquisition and command potentials were controlled using pCLAMP 10.5 software (Axon Instruments). All electrophysiological experiments were conducted at room temperature (25 ± 1°C).

The voltage dependence of ion channel activation was determined from the I/I_max_ ratio by using the *I*‐*V* relationship. The voltage dependence of the steady‐state inactivation relationship was examined by using a standard 2‐pulse protocol. Inactivation variability (I/I_max_) was determined as current at a given pre‐pulse potential divided by the maximum current. The relationship between I/I_max_ and membrane potential was fitted to the Boltzmann equation: I/I_max_ = 1/ {1 + exp [(V_1/2_ − V) / K]}, where V_1/2_ is the estimated half‐maximum activation or inactivation voltage and K is the slope factor. Time‐dependent recovery of ion channels from inactivation was studied using a paired‐pulse protocol (P_1_, P_2_). Current during P_2_ (I_2_) relative to the current during P_1_ (I_1_) was plotted as a function of the P_1_‐P_2_ interval. The time course of recovery fitted well to an exponential function. SigmaPlot 10.0 was used to export ion channels current and action potential graphs, GraphPad Prism 8.0 was used to record ion channels current density, resurrection curve, inactivation curve as well as calculation of action potential amplitude (APA), repolarization 50%, 90% of potential duration (APD_50_, APD_90_).

### 4.6 Senescence‐Associated β‐Galactosidase staining

SA‐β‐Gal staining was performed according to the manufacturer's protocol (9860, CST). In brief, cells were washed with 1 × PBS and fixed in β‐galactosidase fixation solution (2% formaldehyde/0.2% glutaraldehyde in 1 × PBS) for 15 min after the culture medium was discarded, and cells were stained in SA‐β‐gal staining solution (pH 6.0) overnight at 37°C. The number of positive SA‐β‐gal staining was determined by inverted bright field microscopy (Nikon, Japan).

### 4.7 Small interfering RNA(siRNA)

siRNA was transfected into cells using Lipofectamine 3000 transfection kit (L3000‐015, Invitrogen), at a final concentration of 50 nM. Furthermore, both plasmid and liposome diluent solutions were completely mixed and incubated at room temperature for 15 min, then added to 1–2 ml Clay medium (containing 10% FBS) dropwise. The cells were incubated in an incubator at 37°C and 5% CO_2_. Fresh medium (10%FBS) was replaced on the six‐well plates after 24 h, next, AGEs or anti‐RAGE antibody were added and cultured for 48 h, aiming to explore the mechanism between diabetes and senescence.

siRNA‐p16, siRNA‐Rb, and their negative controls were designed and synthesized by Guangzhou GeneYuan Biotechnology Co. Ltd. (Guangzhou, China). The sequences of siRNA‐p16 and siRNA‐Rb used in this study were shown in Table S5, respectively. Finally, the inhibitory efficiency of siRNA was verified by Western blot.

### 4.8 Western blot analysis

Atrial tissue and HL‐1 cells were both homogenized in RIPA lysis buffer (20–188, Millipore) with Protease/Phosphatase Inhibitor Cocktail (5872, CST). After centrifuged at 12,000 rpm for 15 min at 4°C, the supernatants were collected, and protein quantification was determined by Bicinchoninic Acid (BCA) Protein Assay Kit (P0009, Beyotime, China). Samples were diluted with 4 × loading buffer (9173, TaKaRa, Japan) and heated at 100°C for 10 min. The proteins (30 μg) were fractionated on 10% SDS–polyacrylamide gels and transferred to PVDF membranes (IPVH00010, Millipore) according to the protocols. Membranes were blocked with 5% skim milk (232,100, BD Biosciences) in Tris‐buffered saline Tween (TBST) (AR0031, Boster) for 1 h at room temperature before overnight incubation at 4°C with the primary antibodies including rabbit polyclonal to Cav1.2 (1:1000; Alomone; ACC‐003); rabbit polyclonal to Kv1.5 (1:1000; APC‐004; Alomone); rabbit polyclonal to Kv4.3 (1:1000; APC‐017; Alomone); rabbit polyclonal to p16 (1:1000; Abcam; UK; ab189034); rabbit polyclonal to Rb (1:1000; ab226979; Abcam, UK); rabbit polyclonal to AGE (1:1000; ab51995; Abcam, UK); rabbit polyclonal to RAGE (1:1000; ab65965; Abcam, UK). The signals were normalized to the protein levels of glyceraldehyde 3‐phosphate dehydrogenase (GAPDH) (1:10,000; 60,004‐l‐lg; Proteintech). After washing in TBST, the membranes were incubated for 1 h with horseradish peroxidase (HRP)‐conjugated anti‐rabbit IgG (7074 CST) or anti‐mouse IgG (7076; CST) in 5% skim milk. Protein bands were visualized using electrochemiluminescence (ECL) reagents (BL520A; Biosharp) and exposure machine (ImageQuant LAS500). The films were evaluated densitometrically using the public domain ImageJ software (National Institutes of Health). If necessary, the membranes were stripped and re‐probed using stripping buffer (21,059, Thermo Fisher Scientific).

### 4.9 Cell cycle profiling

The Cell Cycle Detection Kit (Beckman Coulter, Brea, CA) was used for cell cycle analysis. Cells were collected in a centrifuge tube, washed twice with 1 × PBS, then 300 μl PBS was added to resuspend the cells, 700 μl anhydrous ethanol was added dropwise with a vortex vibrator, then fixed at −20°C for 4 h. The supernatant was centrifuged and washed twice with PBS, added with BD Pharmingen™ PI/RNase staining buffer staining solution (550,825; BD Biosciences) and incubated at room temperature and protection from light for 15 min, then the cell suspension was passed through a 200 μm screen into a flow tube, finally, the cell suspension was detected by Flow cytometry (CytoFlex, Beckman‐Coulter). These results were analyzed by Modifit software.

### 4.10 Statistical analysis

Statistical tests were performed using SPSS 20.0 (IBM, Armonk, NY). The two‐tailed Student's *t*‐test or one‐way analysis of variance followed by the Newman–Keuls test was used to analyze the data from two groups or more than two groups, respectively. All data are expressed as mean ± S.E.M. *p* < 0.05 was regarded as statistically significance.

## AUTHOR CONTRIBUTIONS

C. Deng and H. Yang conceived and designed the research. D. Zheng, Q. Wu, and P. Zeng performed the experiments, collected the data, analyzed the data, and wrote the manuscript. S. Li, Y. Cai, S. Chen, and X. Luo contributed to performing the research and preparing experimental reagents. S. Kuang, F. Rao, Y. Lai, M. Zhou, and F. Wu helped to analyze the data and provide the materials. All authors read and approved the final manuscript.

## CONFLICT OF INTEREST

The authors declare that they have no conflict of interest. All institutional and national guidelines for the care and use of laboratory animals were followed.

## Supporting information


**Appendix S1** Supporting Information

## Data Availability

The data sets used and/or analyzed during the current study are available from the corresponding author on reasonable request.
