# Supplementary material for: Advanced glycation end products induce senescence of atrial myocytes and increase susceptibility of atrial fibrillation in diabetic mice
Source: Aging Cell. 2022 Oct 24;21(12):e13734. doi: 10.1111/acel.13734 (PMC9741501; doi:10.1111/acel.13734)
Supplement: Supplementary file 1 — Appendix S1 Supporting Information [file ACEL-21-e13734-s001.docx]

**Supplemental information**

**
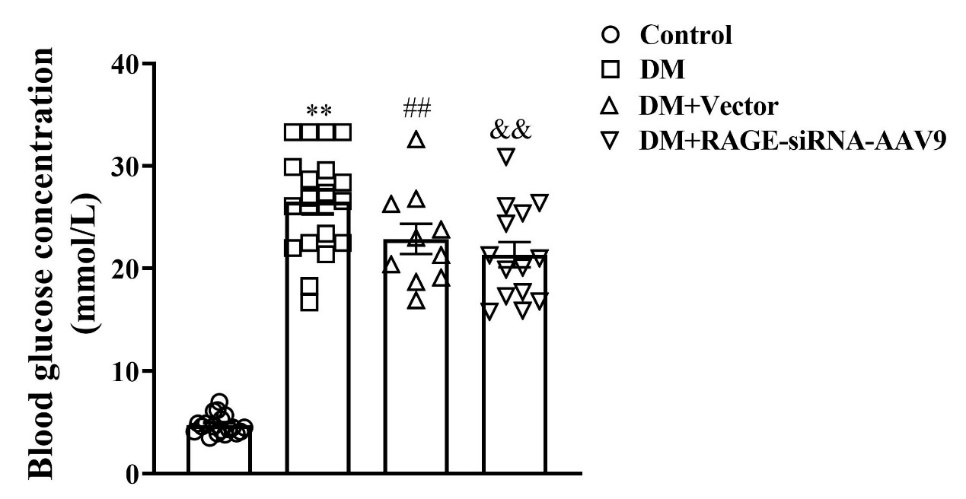
**

**Figure S1. Changes in random glucose level after streptozotocin (STZ) or RAGE-siRNA-AAV9 injection in C57/BL6 mice.** The changes of random blood glucose concentration (mmol/L) in the Control (*n* = 19), DM (*n* = 19), DM + Vector (*n* = 10), DM + RAGE-siRNA-AAV9 (*n* = 14) groups. Data are presented as mean ± SEM; ^**^*P* < .01, ^##^*P* < .01, ^&&^*P* < .01 *vs.* Control group.


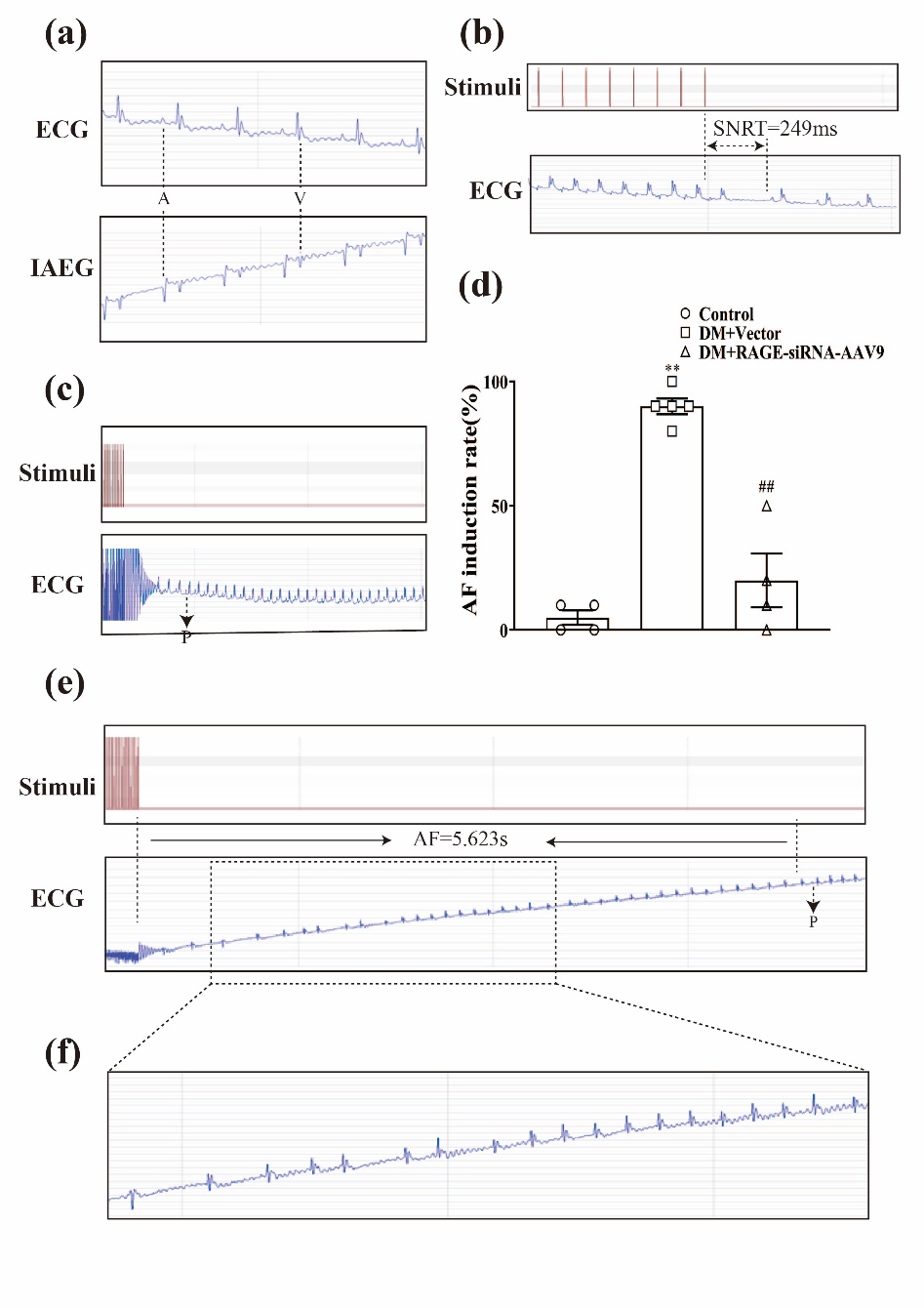


**Figure S2. Representative electrophysiology results for atrial rapid pacing in diabetic mice with or without intervention with RAGE-siRNA-AAV9**. (a) Typical baseline surface electrocardiogram (ECG) and intra atrial electrocardiogram (IAEG). (b) Typical surface ECG recordings of sinus node recovery time (SNRT) following a 6s pacing train. (c) Typical surface ECG recordings of rats maintaining SR after 15 s of atrial burst pacing. (d) AF induction rate in Control, DM + Vector and DM + RAGE-siRNA-AAV9 mice (*n* = 4-5). (e) Typical surface ECG recordings of DM mice with AF that spontaneously reverted to SR. (f) Typical disorganized atrial wave (f wave). ^**^*P* < .01 *vs.* Control group. ^##^*P* < .01 *vs.* DM + Vector group.


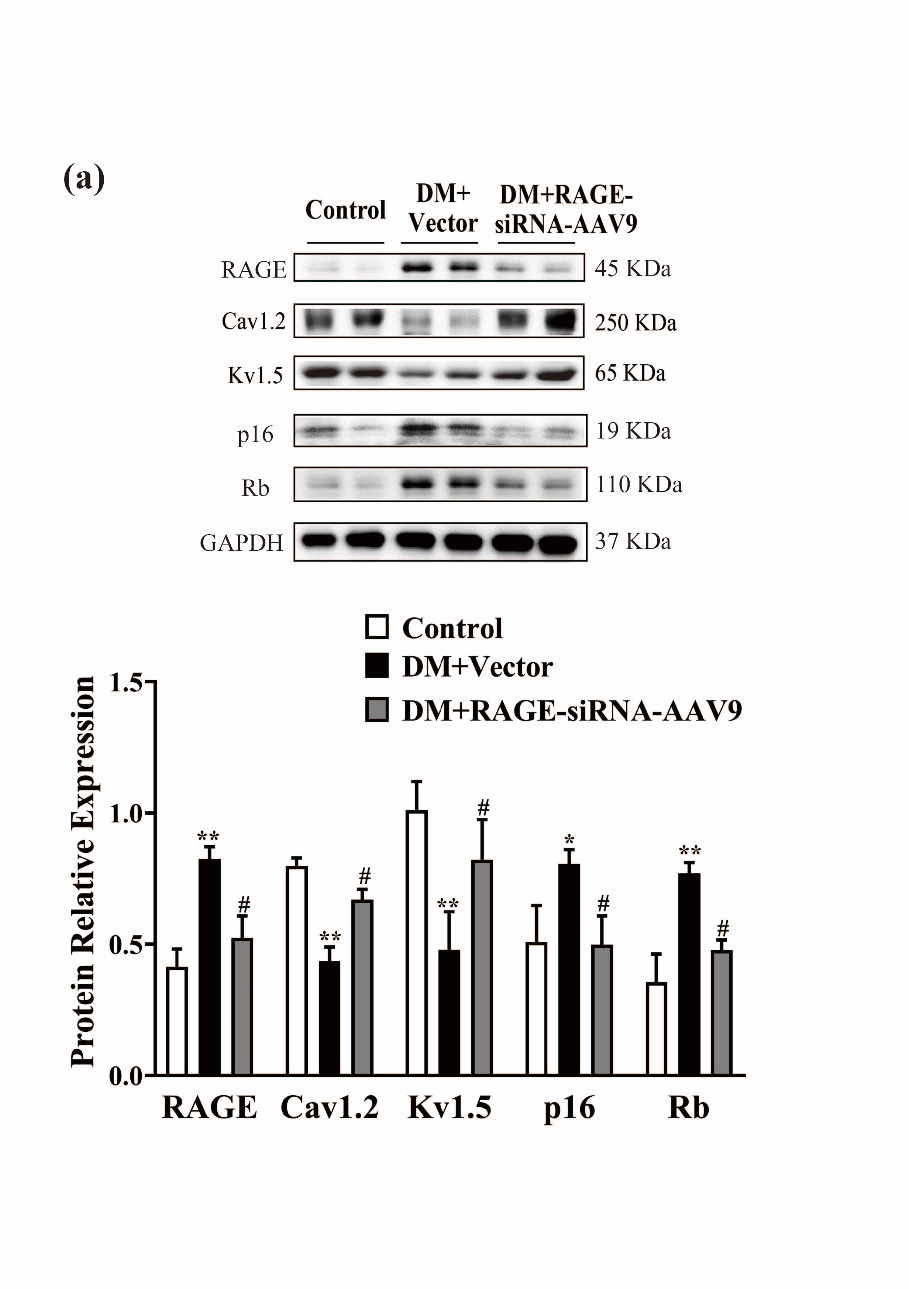


**Figure S3. Expression of RAGE, ion channels and p16, Rb proteins in diabetic mice with or without intervention with RAGE-siRNA-AAV9.** (a) Representative blots and densitometry analysis of RAGE, ion channels, p16 and Rb proteins in atrial tissues from Control, DM + Vector and DM + RAGE-siRNA-AAV9 groups (*n* =4-6). ^*^*P* < .05, ^**^*P* < .01 *vs.* Control group, ^#^*P* < .05 *vs.* DM + Vector group.

**Table S1. Susceptibility to AF and related electrophysiological characteristics in Control and Diabetic mice.**

Data are presented as mean ± SEM; ^*^*P* < .05, ^**^*P* < .01 *vs.* Control group. AFIR: AF induction rate; MDAF: mean duration of AF; PWD: P wave duration; SCL: sinus cycle length; SNRT: sinus node recovery time; CSNRT: corrected sinus node recovery time; PR: PR interval; QRS: QRS interval; QT: QT interval.

|  | Control (*n* = 7) | DM (*n* = 7) |
| --- | --- | --- |
| AFIR (%) | 9.00 | 93.00^**^ |
| MDAF (s) | 0.78 ± 0.29 | 3.48 ± 0.63^**^ |
| SCL (ms) | 138.27 ± 7.54 | 163.79 ± 5.17^*^ |
| SNRT (ms) | 175.71 ± 9.31 | 216.00 ± 26.66 |
| CSNRT (ms) | 32.71 ± 2.78 | 64.31 ± 17.78 |
| PWD (ms) | 18.29 ± 1.21 | 22.57 ± 1.25^*^ |
| PR (ms) | 38.43 ± 1.00 | 42.43 ± 1.46 |
| QRS (ms) | 12.71 ± 0.29 | 14.00 ± 0.62 |
| QT (ms) | 28.14 ± 0.46 | 28.71± 0.42 |

**Table S2.** **Effects of Action potential duration and *I_Ca,L_, I_to_* of atrial myocytes in atriums of diabetic mice.**

Data are presented as mean ± SEM; ^**^*P* < .01 *vs.* Control group. a: activation; i: inactivation; *S*: slope; *V_1/2_*: Half activation/half inactivation voltage; *τ*: time constant; APA: action potential amplitude; APD_50_: action potential when repolarization 50% procedures; APD_90_: action potential when repolarization 90% procedures.

|  | Control (*n* = 9-16) | DM (*n* = 10-15) |
| --- | --- | --- |
| APA (mV) | 89.52 ± 3.39 | 94.08 ± 2.92 |
| APD_50_ (ms) | 16.65 ± 2.27 | 33.52 ± 3.92^**^ |
| APD_90_ (ms) | 57.74 ± 36.6 | 133.56 ± 6.70^**^ |
| *S*_a_ of *I_Ca,L_* | 8.35 ± 0.59 | 8.63 ± 1.17 |
| *V*_a-1/2_ of *I_Ca,L_* | -12.54 ± 1.35 | -14.30 ± 1.80 |
| *S*_i_ of *I_Ca,L_* | -5.88 ± 0.44 | -8.17 ± 0.61^**^ |
| *V*_i-1/2_ of *I_Ca,L_* | -35.79 ± 1.03 | -42.11 ± 1.63^**^ |
| τ of *I_Ca,L_* | 86.41 ± 16.47 | 93.73 ± 9.93 |
| *S*_a_ of *I_to_* | 21.55 ± 0.06 | 18.25 ± 1.34 |
| *V*_a-1/2_ of *I_to_* | 21.19 ± 3.69 | 22.11 ± 2.93 |
| *S*_i_ of *I_to_* | -4.37 ± 0.37 | -8.52 ± 0.84^**^ |
| *V*_i-1/2_ of *I_to_* | -36.45 ± 2.88 | -37.10 ± 1.17 |
| τ of *I_to_* | 48.34 ± 11.98 | 30.6 ± 2.76 |

**Table S3. Effects of AGEs and RAGE on Action potential duration and *I_Ca,L_* in HL-1 cells.**

Data are presented as mean ± SEM; ^**^*P* < .01 *vs.* BSA group; ^##^*P* < .01 *vs.* AGEs group. a: activation; i: inactivation; *S*: slope; *V_1/2_*: Half activation/half inactivation voltage; τ: time constant; APA: action potential amplitude; APD_50_: action potential when repolarization 50% procedures; APD_90_: action potential when repolarization 90% procedures.

|  | BSA (*n* = 7-12) | AGEs (*n* = 6-8) | AGEs + anti-RAGE  (*n* = 7-12) |
| --- | --- | --- | --- |
| APA (mV) | 86.36 ± 5.70 | 100.29 ± 6.35 | 89.93 ± 1.09 |
| APD_50_ (ms) | 6.05 ± 0.49 | 16.78 ± 2.33^**^ | 5.62 ± 0.23^##^ |
| APD_90_ (ms) | 25.32 ± 1.62 | 64.13 ± 3.13^**^ | 28.45 ± 0.75^##^ |
| *S*_a_ of *I_Ca,L_* | 5.65 ± 0.36 | 7.87 ± 1.30 | 5.95 ± 0.51 |
| *V*_a-1/2_ of *I_Ca,L_* | -13.78 ± 1.60 | -14.97 ± 3.24 | -15.35 ± 1.49 |
| *S*_i_ of *I_Ca,L_* | -13.14 ± 1.59 | -11.78 ± 1.19 | -15.73 ± 3.13 |
| *V*_i-1/2_ of *I_Ca,L_* | -59.73 ± 3.72 | -57.91 ± 6.84 | -62.45 ± 6.03 |
| τ of *I_Ca,L_* | 184.59 ± 25.49 | 159.89 ± 11.49 | 201.48 ± 20.15 |

**Table S4. Susceptibility to AF and related electrophysiological characteristics in Control, DM + Vector and DM + RAGE-siRNA-AAV9 mice.**

Data are presented as mean ± SEM; ^**^*P* < .01 *vs.* Control group, ^##^*P* < .01 *vs.* DM + Vector group. AFIR: AF induction rate; MDAF: mean duration of AF; PWD: P wave duration; SCL: sinus cycle length; SNRT: sinus node recovery time; CSNRT: corrected sinus node recovery time; PR:PR interval; QRS: QRS interval; QT: QT interval.

|  | Control (*n* = 4) | DM + Vector (*n* = 5) | DM + RAGE-siRNA-AAV9 (*n* = 4) |
| --- | --- | --- | --- |
| AFIR (%) | 5.00 | 90.00^**^ | 20.00^##^ |
| MDAF (s) | 0.83 ± 0.48 | 4.07 ± 0.42^**^ | 1.43 ± 0.48^##^ |
| SCL (ms) | 178.68 ± 6.15 | 161.16 ± 12.38 | 117.3 ± 3.59^##^ |
| SNRT (ms) | 234.5 ± 20.17 | 265 ± 9.47 | 180 ± 23.26^##^ |
| CSNRT (ms) | 61.23 ± 6.09 | 111.54 ± 20.28^**^ | 58.1 ± 17.03^##^ |
| PWD (ms) | 19.25 ± 1.93 | 18.6 ± 0.75 | 16.5 ± 0.65 |
| PR (ms) | 45.25 ± 2.17 | 42.6 ± 0.93 | 41.25 ± 2.25 |
| QRS (ms) | 13.5 ± 0.65 | 12.8 ± 0.37 | 13.5 ± 0.29 |
| QT (ms) | 30.5 ± 1.50 | 27.4 ± 0.40 | 27.5 ± 0.65 |

**Table S5. Sequences of siRNA oligonucleotides**.

| **Target** | **Sequence (5' → 3')** |  |
| --- | --- | --- |
|  | **Sense strand** | **Antisense strand** |
| mRb1 | GCAUAGAAACCAGUGUCUATT | UAGACACUGGUUUCUAUGCTT |
| mCdkn2a | CGUGUCUAGCAUGUGGCUUUAAAAA | UUUAAAGCCACAUGCUAGACACGCT |
| RAGE-siRNA-AAV9 | accggGGGCATTCAGCTGTTGGTTGAttcaagagaTCAACCAACAGCTGAATGCCCttttt | tctaaaaaaGGGCATTCAGCTGTTGGTTGAtctcttgaaTCAACCAACAGCTGAATGCCCc |
